# Aminergic Signaling Controls Ovarian Dormancy in *Drosophila*

**DOI:** 10.1038/s41598-018-20407-z

**Published:** 2018-02-01

**Authors:** Gabriele Andreatta, Charalambos P. Kyriacou, Thomas Flatt, Rodolfo Costa

**Affiliations:** 1 Department of Biology, University of Padova, Padova, Italy; 2 Department of Genetics, University of Leicester, Leicester, United Kingdom; 3 Department of Ecology and Evolution, University of Lausanne, Lausanne, Switzerland; 4 Department of Biology, University of Fribourg, Fribourg, Switzerland

## Abstract

In response to adverse environmental conditions many organisms from nematodes to mammals deploy a dormancy strategy, causing states of developmental or reproductive arrest that enhance somatic maintenance and survival ability at the expense of growth or reproduction. Dormancy regulation has been studied in *C. elegans* and in several insects, but how neurosensory mechanisms act to relay environmental cues to the endocrine system in order to induce dormancy remains unclear. Here we examine this fundamental question by genetically manipulating aminergic neurotransmitter signaling in *Drosophila melanogaster*. We find that both serotonin and dopamine enhance adult ovarian dormancy, while the downregulation of their respective signaling pathways in endocrine cells or tissues (insulin producing cells, fat body, *corpus allatum*) reduces dormancy. In contrast, octopamine signaling antagonizes dormancy. Our findings enhance our understanding of the ability of organisms to cope with unfavorable environments and illuminate some of the relevant signaling pathways.

## Introduction

Dormancy is a widespread phenotypically plastic response that enables organisms – both vertebrates and invertebrates – to survive adverse environmental conditions^1–3^. Two types of dormancy can be distinguished. Quiescence is a direct, immediate response to unfavorable conditions; it may be facultative or inevitable and can be adaptive or not. In contrast, diapause represents an adaptive response to anticipatory token cues (e.g., temperature, photoperiod) in unfavorable, predictable environments and includes defined physiological phases (prediapause, diapause, postdiapause). Both responses profoundly alter metabolism, increase stress tolerance, and promote survival ability, and have been studied in the nematode *C. elegans* as well as in various insects^4–9^. Several endocrine signaling pathways have been implicated in inhibiting dormancy under normal conditions, but little is known about the mechanisms that lead to the induction of dormancy by suppressing these pathways^1,2,10^.

In fruit flies (*Drosophila*) and other insects, adult females enter ovarian dormancy by arresting gonadal maturation in response to cold (<13 °C; enhanced by shorter photoperiods), a state that is accompanied by markedly improved stress resistance, slowed aging and increased adult survival^5,9,11–16^. This response involves the downregulation of several gonadotropic hormones, including juvenile hormone (JH, produced in the *corpus allatum* [CA] gland), 20-hydroxy-ecdysone (20E; produced in the ovary), and insulin-like peptides (ILPs = dILPs in *Drosophila*; produced in insulin-producing cells [IPCs] in the brain)^1,5,9,15,17^. For instance, dormant fruit flies, monarch butterflies and mosquitos exhibit reduced JH and/or 20E levels^18–21^, and dormancy can be terminated by topical application of JH or 20E^11,12,19,21–23^. Similarly, surgical or genetic ablation of the CA in grasshoppers, butterflies and fruit flies^4,24,25^ and genetic downregulation of components of insulin/insulin-like signaling (IIS) in *Drosophila*^12,26^ faithfully phenocopy major aspects of dormancy, including halted or decreased reproduction, improved stress resistance and greatly extended lifespan.

In support of a direct role of the IIS pathway in regulating dormancy in *Drosophila*, we have recently shown that two insulin-like peptides, dILP2 and dILP5, produced in the IPCs, act as major endocrine antagonists of dormancy^15^ (also see^14^). Since the IPCs adjust insulin release in response to environmental inputs (e.g., cold, nutrients)^27–29^, and because IIS is required for the production of both JH and 20E^12,30–32^, these cells likely represent a critical target of dormancy-inducing cues. Yet, how neurosensory signals relay dormancy-inducing cues to the IPCs and to peripheral tissues (e.g., CA, fat body, ovary) in order to control dormancy is poorly understood; neurotransmitter signaling might represent an attractive candidate mechanism in this context.

Indeed, previous data suggest that aminergic neurotransmitters, such as the biogenic amines serotonin, dopamine or octopamine, might be involved in regulating dormancy, yet the evidence to date is largely correlational^10,33–36^. For example, these neurotransmitters regulate many processes that are also known to be affected by dormancy, including development, reproduction, behavior, stress, immunity and aging^37–45^. Notably, diapausing butterflies and moths exhibit elevated levels of serotonin and dopamine^33,35,36,46^. Moreover, biogenic amines can affect the production and/or release of hormones involved in dormancy regulation^47^. The expression and/or release of dILPs, for instance, is modulated by aminergic receptors for serotonin (5-hydroxytryptamine receptor 1 A = 5-HT1A)^48,49^, GABA (metabotropic γ-aminobutyric acid (GABA)-B receptor 2 = GBR)^29,50^, and octopamine (octopamine receptor in mushroom bodies = OAMB)^49,51^. Aminergic signaling also regulates the levels of JH and 20E, thus potentially modulating how these hormones regulate dormancy^52–57^. However, whether aminergic signaling causally regulates insect dormancy has not yet been directly investigated.

Here we use neurogenetic manipulations in *Drosophila melanogaster* to study whether aminergic signaling via the serotonin, octopamine, GABA, and dopamine neurotransmitters regulates reproductive dormancy. Since in *D. melanogaster* dormancy is rapidly induced and weak, lacking clear preparatory and postdiapause phases, some authors argue that it might represent quiescence rather than diapause; we thus prefer to use the neutral term ‘dormancy’. By downregulating aminergic signaling we identify neurotransmitters that either positively or negatively regulate the phenotype. Our results demonstrate that both serotonin and dopamine promote ovarian dormancy by relaying dormancy-inducing cues to major endocrine sites known to be central for dormancy physiology, including the IPCs, CA and fat body. Octopaminergic signaling, by contrast, acts to antagonize dormancy entry. Together, our results provide functional evidence for a major role of aminergic signals in modulating dormancy. These findings add to our understanding of how organisms can adjust growth, reproduction, somatic maintenance and survival in response to adverse environmental conditions.

## Results

### Serotonin promotes dormancy by regulating insulin signaling

Insulin/insulin-like growth factor signaling (IIS) is thought to play a major role in dormancy regulation but whether neurotransmitters can modulate this control is largely unclear. Since dILPs act as dormancy antagonists^14,15^, and because the production of dILPs by the IPCs is inhibited by the serotonin receptor^48,49^, we first asked whether manipulating serotonin receptor levels in the IPCs affects dormancy.

**Figure 1:**
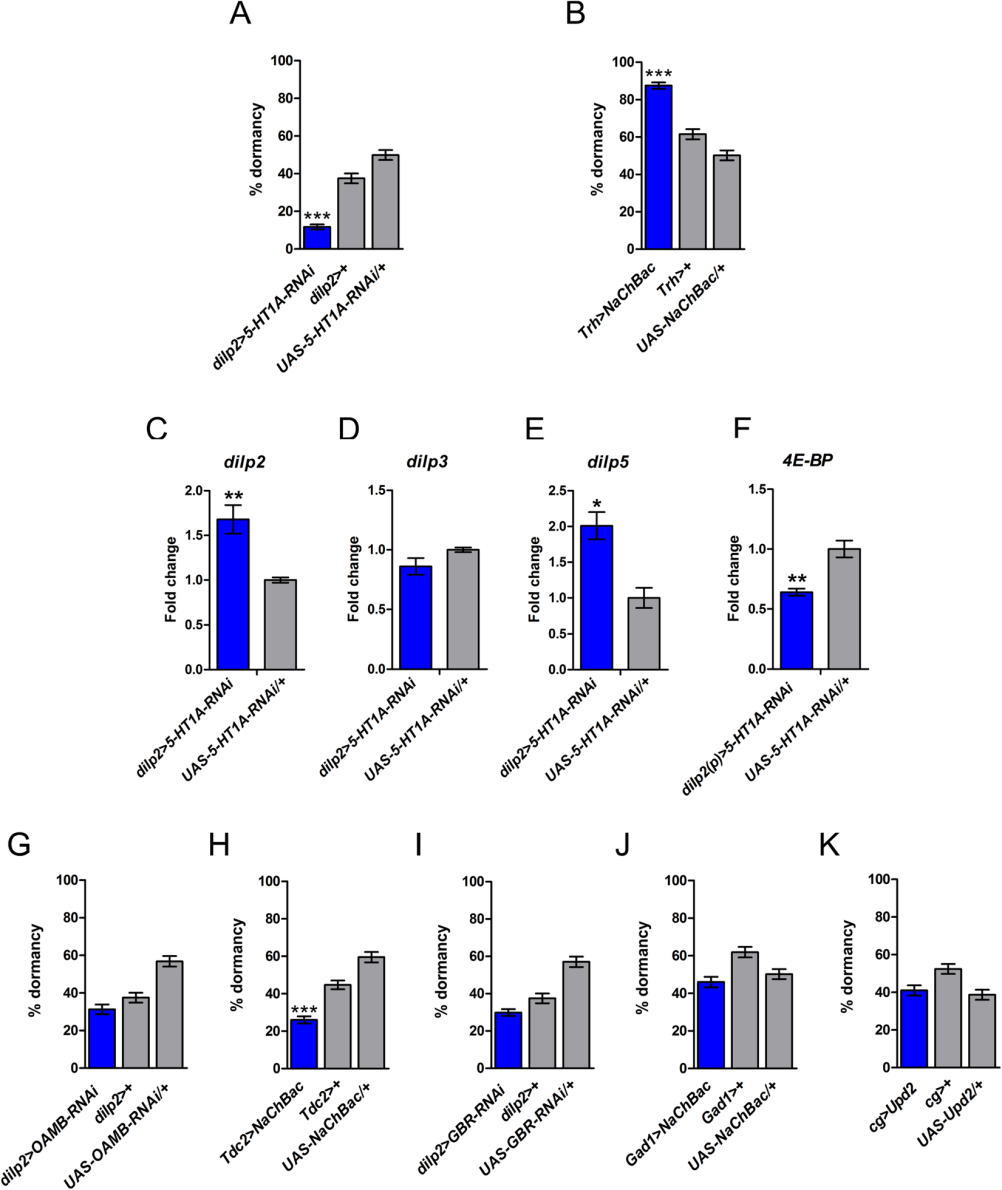
Serotonergic signaling promotes dormancy regulating IIS, whereas octopamine inhibits dormancy. (**A**) Knocking down the serotonin receptor *5-HT1A* in the IPCs strongly reduces dormancy levels (cf. Supplementary Figure S1A), suggesting that serotonin promotes dormancy. (**B**) Constitutive activation of serotoninergic neurons for 11 days at 12 °C and 16 h:8 h L:D increases ovarian dormancy, confirming that serotonin is a positive regulator of dormancy. Figure 1A and B show percentage dormancy (mean ± binomial SE); assays were performed with 5–7 replicates per genotype, each replicate consisting of ~60 females. ***p < 0.001. (**C–F**) Expression levels (mean ± standard error [SE]) of *dilp2*, *dilp3*, *dilp5* in *dilp2* > *5-HT1A-RNAi* and *4E-BP* in *dilp2(p)* > *5-HT1A-RNAi* females at 12 °C as compared to controls. *p < 0.05; **p < 0.01. (**G**) Downregulation of octopaminergic signaling via *OAMB* RNAi in the IPCs does not affect dormancy (also see Supplementary Figure S1B). (**H**) In contrast, however, constitutive activation of octopaminergic neurons decreases dormancy levels. (**I**) Knockdown of *GBR* in the IPCs with *dilp2*-GAL4 has no effect on dormancy (but see Supplementary Figure S1C for a positive yet inconsistent result with *dilp2(p)*-GAL4). (**J**) In support of the notion that GABA does not affect dormancy, constitutive activation of GABAergic neurons is ineffective in regulating dormancy. (**K**) Overexpression of *Upd2* in fat body with *cg-*GAL4, a manipulation that blocks GABA-mediated inhibition of dILP release from the IPCs, does not affect dormancy (also see Supplementary Figure S1D). Shown are the percentage of females in dormancy (mean ± binomial SE); assays were performed with 5–10 replicates per genotype, each replicate consisting of ~50 females. ***p < 0.001.

To address this question, we downregulated the *5-HT1A* serotonin receptor in the IPCs using RNAi under the control of two different IPC-specific GAL4 drivers (*dilp2(p)-Gal4*^58,59^; *dilp2-Gal4*^60–62^). Knockdown of the *5-HT1A* receptor in the IPCs strongly suppressed the dormancy response (Fig. 1A and Supplementary Figure S1A), thus lending support to the notion that elevated IIS acts to inhibit dormancy^15^.

To determine whether upregulation of serotonin signaling would have the opposite effect, we constitutively activated serotonergic neurons by overexpressing a low-threshold, voltage-gated bacterium sodium channel *NaChBac* under the control of a *tryptophan hydroxylase* (*TRH*)-GAL4 line which drives expression in 5HT-positive neurons (*TRH*-GAL4 > UAS-*NaChBac*^63,64^). The resulting hyperexcitation of serotonergic neurons significantly increased the proportion of females in reproductive dormancy (Fig. 1B), thus confirming that serotonin represents a positive regulator of dormancy, presumably by reducing IIS.

To examine whether serotonin signaling might regulate dormancy by modulating IIS, we measured dILP mRNA levels upon manipulation of the serotonin receptor in the IPCs. In agreement with previous data showing that the *5-HT1A* serotonin receptor inhibits dILP production under normal (non-dormancy) conditions^49^, we found that under dormancy conditions RNAi against the *5-HT1A* receptor in the IPCs causes a derepression (upregulation) of *dilp2* and *dilp5* but not of *dilp3* (Fig. 1C–E).

To further corroborate that serotonin signaling affects dormancy via IIS we assayed the expression levels of a major transcriptional target of the IIS/TOR (Target Of Rapamycin) pathway, the translation inhibitor *4E-BP*, outside the brain. Reduced activity of the IIS/TOR pathway results in nuclear translocation of the forkhead box O transcription factor FOXO, whose activation in turn induces *4E-BP*^65^. In line with the observed upregulation of *dilp2* and *dilp5*, we found that knockdown of the *5-HT1A* receptor in the IPCs reduces expression of *4E-BP* throughout the body (thorax and abdomen) (Fig. 1F), in agreement with reduced systemic IIS. These results indicate that dormancy is under serotonergic control via the inhibition of dILP production and release by the serotonin receptor, leading to reduced IIS and promoting entry into the dormant state.

### Octopamine inhibits dormancy but GABA has no effect

We next investigated whether also octopaminergic and GABAergic signaling might contribute to the regulation of dormancy induction. Both octopaminergic and GABAergic signaling are known to influence the production and/or release of hormones involved in dormancy regulation, i.e. dILPs^29,49–51,66^ as well as ecdysone^52,53,67^, so these neurotransmitters might affect dormancy via modulating these hormones.

Silencing of the *octopamine receptor in mushroom bodies* (*OAMB*) in the IPCs had no effect on dormancy levels (Fig. 1G and Supplementary Figure S1B), yet activation of octopaminergic neurons with a *tyrosine decarboxylase 2* (*TDC2*)-GAL4 driver (*TDC2*-GAL4 > UAS*-NaChBac*) decreased dormancy levels (Fig. 1H). Thus, while *OAMB* RNAi did not affect dormancy, the fact that increased octopaminergic signaling reduces dormancy suggests that octopamine likely represents an antagonist that is downregulated under conditions of dormancy.

In contrast to octopamine signaling, GABAergic signaling did not consistently affect dormancy. Although downregulation of the metabotropic GABA(B) receptor (*GBR*) in the IPCs using the *dilp2(p)-*GAL4 driver decreased dormancy levels (see Supplementary Figure S1C), we could not corroborate this result with a second, independent *dilp2*-GAL4 driver (Fig. 1I). Consistent with the ineffectiveness of GABA in modulating dormancy, constitutive activation of GABAergic neurons with a *glutamic acid decarboxylase* (*GAD1*)-GAL4 driver (*GAD1*-GAL4 > UAS-*NaChBac*) also had no effect on dormancy (Fig. 1J). To further corroborate these negative results we manipulated the leptin-like cytokine *Unpaired2* (*Upd2*), a fat body-secreted factor known to block the GABA-mediated inhibition of dILP release from the IPCs and to enhance systemic IIS^29^. When overexpressing *Upd2* in the fat body under the control of two independent fat body-specific GAL4 drivers, *collagen type IV* (*Col4a1* = *Cg*)*-*GAL4^68^ and *pumpless* (*ppl*)*-*GAL4^69,70^, the percentage of flies in dormancy was unaffected (Fig. 1K and Supplementary Figure S1D). Dormancy entry might thus be regulated by octopaminergic signaling but – interestingly – not by GABAergic signaling, even though GABA is known to impact IIS.

### Dopamine is a positive regulator of dormancy

**Figure 2:**
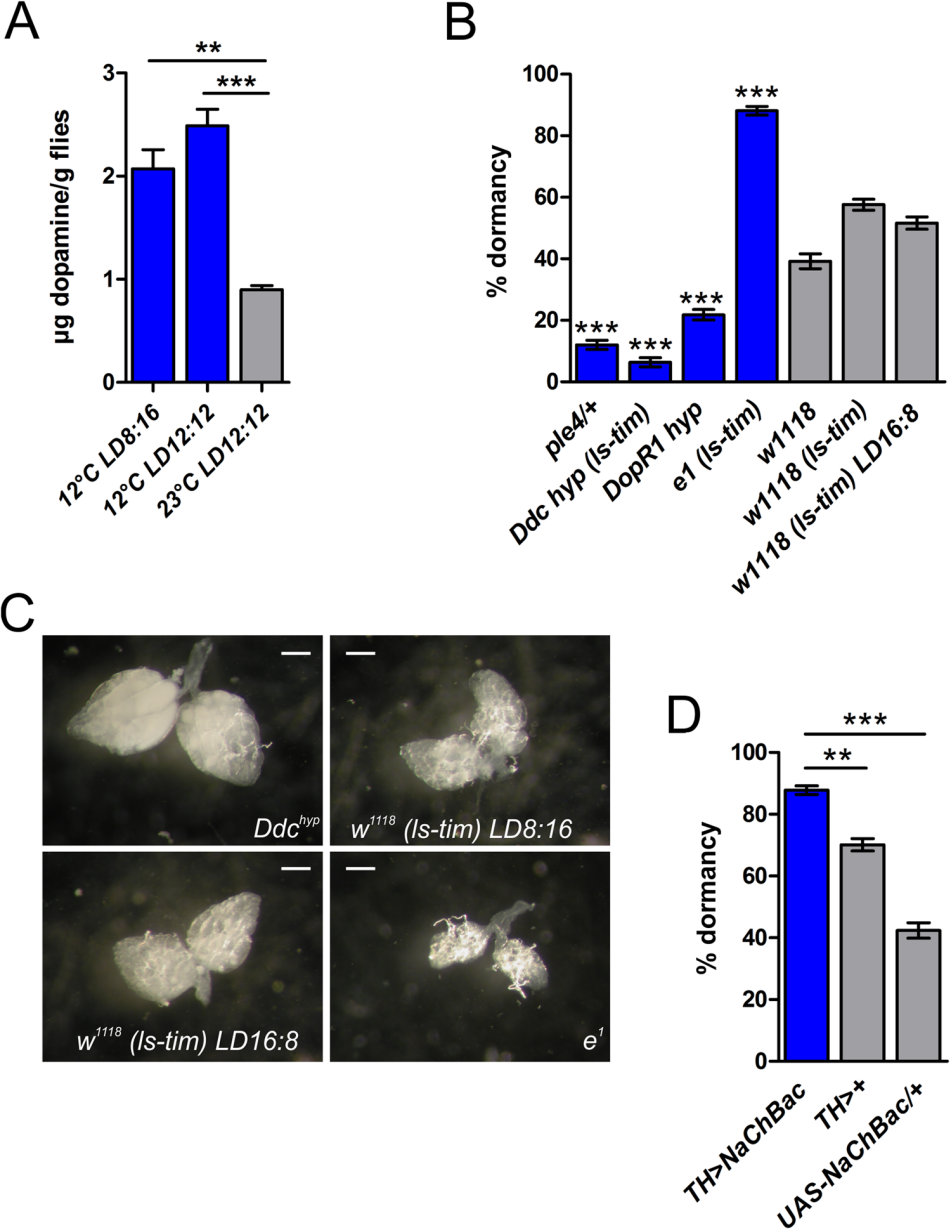
Dopamine is a positive regulator of dormancy. (**A**) Dormancy-inducing conditions (exposure of females to 12 °C and either to 8 h:16 h or 12 h:12 h L:D during 11 days) significantly increases dopamine levels. Shown are mean dopamine levels ± SE (3 replicates per condition, with 500 females each). p-values from *t*-test: **p < 0.01; ***p < 0.001. (**B**) Mutations that impact dopamine synthesis and/or signaling (*ple*^4^, *Ddc*^hyp^, *DopR1*^hyp^) inhibit flies from entering dormancy, whereas *e*^1^ mutant females, which exhibit doubled dopamine levels, show enhanced dormancy. Displayed is the percentage of dormancy (mean ± binomial SE); assays were performed with 4–7 replicates per genotype, each replicate consisting of ~50 females. ***p < 0.001. (**C**) Ovarian development under dormancy conditions in *Ddc*^hyp^ and *e*^1^ mutants as compared to controls. Photographs show representative examples of ovarian development and levels of vitellogenesis after 11 days at 12 °C (scale bars = 0.2 mm). (**D**) Constitutive activation of dopaminergic neurons increases dormancy. Shown is the percentage of dormancy (mean ± binomial SE); assays were performed with 5–6 replicates per genotype, each replicate consisting of ~60 females. **p < 0.01; ***p < 0.001.

In some dormant/diapausing insects the levels of dopamine are elevated, suggesting that dopamine signaling might promote dormancy, yet direct evidence for this regulation is lacking^33–36^. To determine whether – as predicted – dopamine levels are indeed elevated in dormant *D. melanogaster* females we used high performance liquid chromatography (HPLC). Control female flies maintained under dormancy conditions (11 days at 12 °C; with a light [L]:dark [D] cycle of either 8 h:16 h or 12 h:12 h) showed a more than two-fold increase of dopamine titers (2.07 ± 0.32 and 2.49 ± 0.28 µg dopamine/g, respectively) as compared to non-dormant controls raised at 23 °C under long day conditions (0.90 ± 0.07 µg dopamine/g fly) (Fig. 2A), thereby confirming that reduced dopamine is a robust read-out of dormancy state.

To directly test the role of dopamine in dormancy we examined mutant alleles at two loci required for dopamine synthesis: a loss-of-function mutant of *pale* (*ple*), encoding tyrosine-hydroxylase (*ple*^4^/+; see Supplementary Table S1^71–73^), and a hypomorphic mutant of *Ddc*, encoding DOPA-decarboxylase (*Ddc*^hyp^^74,75^). Both mutants exhibited a markedly reduced dormancy response under dormancy-inducing conditions (Fig. 2B), with well advanced ovarian development, as compared to controls (Fig. 2C). Similarly, a hypomorphic mutant of the *Dopamine Receptor 1* (*DopR1*^hyp^^75,76^) showed a significantly reduced dormancy response (Fig. 2B), accompanied by enhanced ovarian development (data not shown).

Since dopamine deficiency decreased dormancy levels in our experiments, we predicted that increased dopamine should promote dormancy entry, as might be expected based on correlational observations in other insect species^33–36^. Indeed, female mutants of *ebony* (*e*^1^), which are characterized by a two-fold increase in dopamine relative to wildtype^77,78^, showed strongly increased dormancy levels, with associated ovarian arrest (Fig. 2B,C). Similarly, dormancy levels were elevated when constitutively activating dopaminergic neurons with a *tyrosine hydroxylase* (*TH*) - GAL4 driver (*TH-*GAL4 > UAS*-NaChBac*) (Fig. 2D). These results thus provide direct evidence that dopamine represents a positive regulator of dormancy state.

### Dopamine promotes dormancy via DopR1 in IPCs, CA and fat body

Previous data suggest that IIS can interact with dopamine signaling and metabolism^79–82^, and two dopamine receptors (DopR1; dopamine 2-like receptor = D2R) are expressed in the CA (the gland producing JH) and fat body (the insect equivalent of mammalian adipose and liver)^57^, two endocrine tissues that are critically important for the physiological regulation of dormancy/diapause in insects^7,8,18,19,24,83^. However, direct evidence for an involvement of dopamine in dormancy control is lacking; we thus aimed to downregulate dopamine receptor signaling at these endocrine sites.

**Figure 3:**
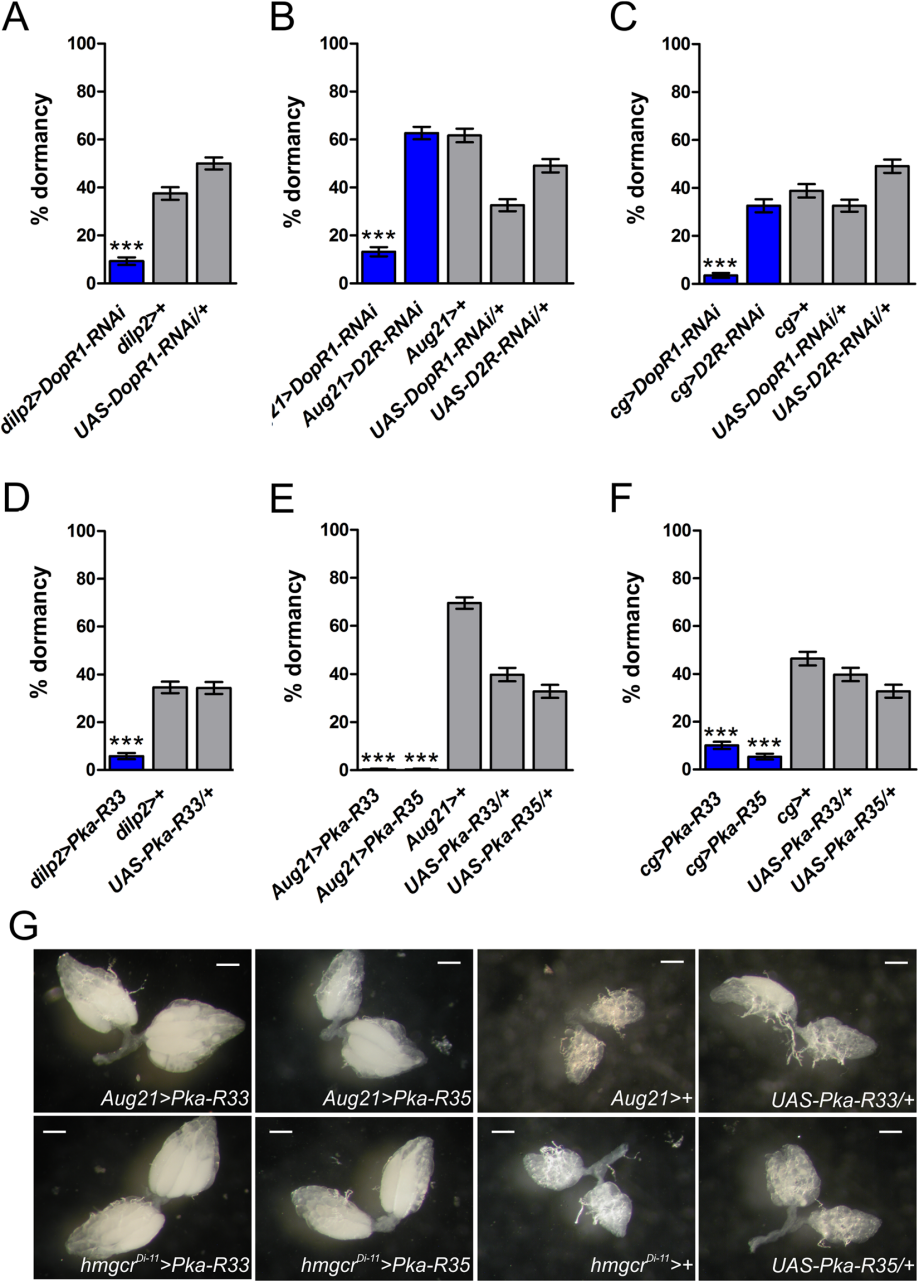
Dopamine promotes dormancy via DopR1 and PKA in IPCs, CA and fat body. (**A**) Knockdown of *DopR1* in the IPCs with *dilp2*-GAL4 reduces dormancy (also see Supplementary Figure S2A). (**B,C**) Downregulation of *DopR1* but not of *D2R* in both CA (**B**) and fat body (**C**) decreases dormancy (also cf. Supplementary Figure S2B,C). (**D–F**) Downregulation of PKA signaling in the IPCs, CA and fat body substantially reduces dormancy levels (also see Supplementary Figure S2D–F). Figure 3A–F show the percentage of females in dormancy (mean ± binomial SE); assays were performed with 5–6 replicates per genotype, each replicate consisting of ~60 females. ***p < 0.001. (**G**) Ovarian development of females expressing mutated forms of *PKA-R* in the CA. Pictures show representative levels of vitellogenesis after 11 days at 12 °C (scale bars = 0.2 mm).

Silencing the *DopR1* receptor in the IPCs markedly decreased dormancy levels (Fig. 3A and Supplementary Figure S2A). Dormancy levels were also strongly reduced when silencing *DopR1* in the CA using two independent CA-specific GAL4 drivers, *Aug21-*GAL4^84^ and *hmgcr*^Di-11^*-*GAL4^85^ (Fig. 3B and Supplementary Figure S2B). In contrast, RNAi directed against *D2R* in the CA had no effect on dormancy levels (Fig. 3B and Supplementary Figure S2B). We obtained equivalent results when we silenced both receptors in the fat body via *cg-*GAL4 or with *ppl-*GAL4: RNAi silencing of *DopR1* decreased dormancy, while *D2R-*RNAi was completely ineffective (Fig. 3C and Supplementary Figure S2C). These observations suggest that signaling via DopR1 – but not via D2R – in the IPCs, CA and fat body contributes to the regulation of dormancy.

### PKA is required in IPCs, CA and fat body for dormancy induction

To further investigate the dopaminergic control of dormancy we manipulated protein kinase A (PKA) signaling: since both DopR1 and D2R converge on PKA^86,87^, dopamine might affect dormancy via PKA signaling. To test this notion, we impaired PKA activity in IPCs, CA and fat body by overexpressing mutated forms of the regulatory subunits of PKA (PKA-R), thereby inhibiting PKA signaling (UAS*-PKA-R33*; UAS-*PKA-R35*). Constitutive inhibition of PKA in the IPCs significantly decreased dormancy levels (Fig. 3D and Supplementary Figure S2D). Likewise, impairing PKA signaling in the CA completely suppressed the dormancy response (Fig. 3E and Supplementary Figure S2E), with females showing advanced vitellogenic stages (Fig. 3G). When expressing both *PKA-R* transgenes in fat body with *cg-*GAL4 or *ppl-*GAL4 we similarly observed strongly reduced dormancy (Fig. 3F and Supplementary Figure S2F). These data indicate that PKA signaling is required in the IPCs, CA and fat body for the proper induction of dormancy.

**Figure 4:**
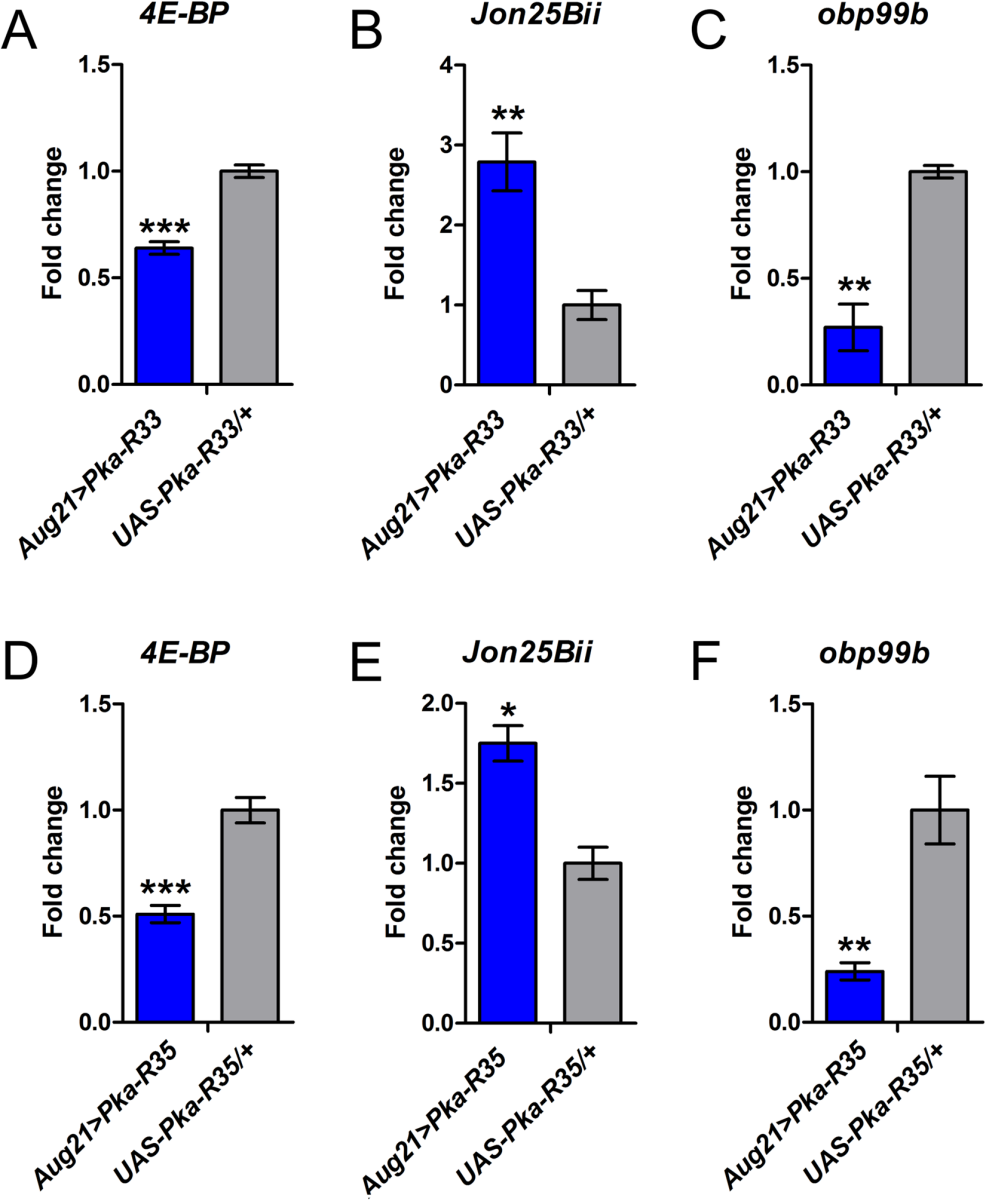
Impaired PKA signaling in the CA alters expression of transcripts involved in IIS and JH signaling. mRNA abundance of transcripts involved IIS and JH signaling upon downregulation of PKA signaling in the CA via expression of mutated forms of *PKA-R* (*PKA-*R33 or *PKA-R35*) which constitutively inhibit PKA. (**A** and **D**) mRNA levels of *4E-BP*, a transcriptional readout of IIS pathway activity; increased IIS is expected to decrease *4E-BP* levels. *Jon25Bii* and *obp99b* are transcriptional readouts of JH signaling; (**B** and **E**) show the mRNA levels of *Jon25Bii*, which is normally induced by JH; and (**C** and **F**) show the mRNA levels of *obp99b*, which is typically downregulated by JH. mRNA levels (mean ± SE) were measured in females kept at 12 °C for 11 days (4 replicates per genotype, each consisting of 10 females). p-values from ANOVA; *p < 0.05; **p < 0.01; ***p < 0.001.

Finally, given that IIS acts in the CA to control JH production^85,88^, and since IIS and JH might be involved in a positive feedback loop^89^, we were interested in measuring transcriptional readouts of IIS and JH signaling in whole bodies from flies expressing PKA-R in the CA under dormancy conditions. High IIS and JH signaling are expected to inhibit dormancy entry (see Introduction). Consistent with increased IIS upon impaired PKA signaling, the levels of *4E-BP* (which is normally inhibited by IIS^65^) were decreased. Since IIS is required for the production of JH, a hormone that inhibits dormancy, we also investigated two transcriptional readouts of JH signaling^25^. Impaired PKA activity caused upregulation of *Jon25Bii*, normally induced by JH, and downregulation of *obp99b*, typically downregulated by JH (Fig. 4A–F), consistent with high JH signaling activity. PKA signaling thus likely affects dormancy via IIS and JH, consistent with the notion that dormancy entry requires the downregulation of both IIS and JH signaling.

## Discussion

**Figure 5:**
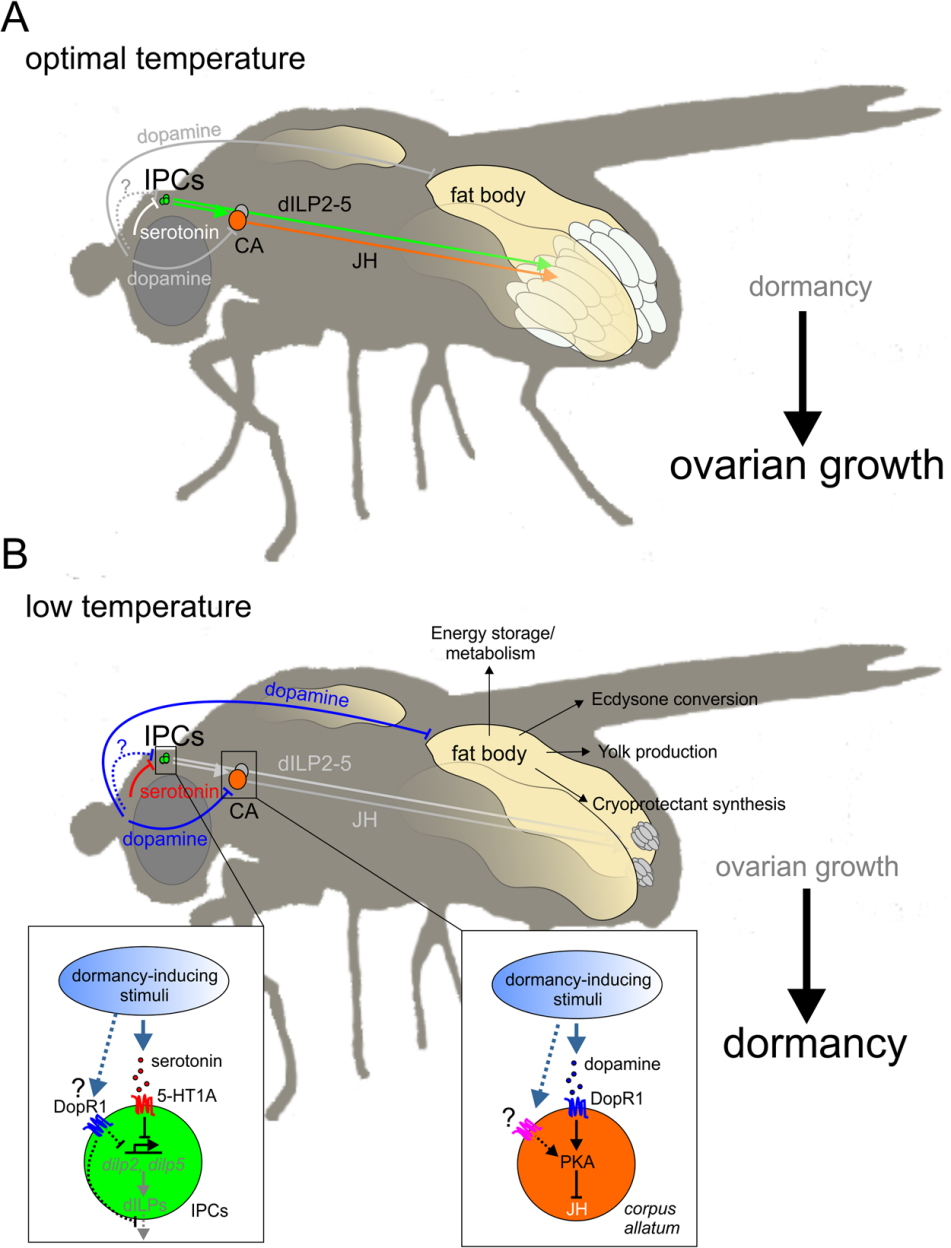
Model of the aminergic signaling control of *Drosophila* dormancy. (**A**) Under normal, non-dormancy conditions, dILPs and JH promote reproduction and ovarian growth at the expense of reduced somatic maintenance; under these conditions, serotonin and dopamine signaling in IPCs, CA and fat body is reduced, thus inhibiting the dormancy response. (**B**) Under dormancy-inducing conditions, in contrast, serotonin and dopamine inhibit the production and/or release of dILPs in the IPCs, thereby causing the downregulation of systemic IIS (and JH signaling) and thus promoting entry into the dormancy state. Similarly, in the CA, dopamine/DopR1 activate PKA signaling which reduces JH synthesis and/or release, thus favoring dormancy induction. Likewise, increased activity of dopaminergic signaling in the fat body promotes reproductive dormancy, perhaps by inhibiting processes required for ovarian maturation (e.g., vitellogenesis). In contrast to serotonin and dopamine, octopamine (not shown in the model) likely represents a dormancy antagonist (see Results). While several of the regulatory connections in this model are hypothetical and remain to be worked out in more detail, our study provides clear evidence that serotonin and dopamine signaling act in key endocrine tissues to promote dormancy entry in *Drosophila*.

Dormancy (i.e., diapause or quiescence) is a physiological state of improved stress resistance and survival ability in response to harsh environmental conditions. Although many physiological studies have examined the hormonal control of this life-history strategy, the identity and effects of neurosensory signals acting upstream of the endocrine system to regulate dormancy are poorly understood. Here we have used neurogenetic manipulations to directly investigate how neurotransmitters regulate dormancy in *Drosophila melanogaster*. Our results show that by acting in endocrine cells and tissues and by modulating hormonal signaling, serotonin and dopamine positively regulate while octopamine counteracts dormancy (Fig. 5) suggesting that these neurotransmitters play opposing roles.

Although serotonin has previously been implicated in dormancy/diapause regulation, direct evidence for its involvement is largely lacking. For example, in the butterfly *Pieris brassicae*, serotonin levels are higher at the beginning of diapause as compared to non-diapausing individuals^35^, and in the moth *Antheraea pernyi* injection of dsRNAi against the serotonin receptor 5-HTRB inhibits diapause^46^. Given that in *Drosophila* the serotonin receptor inhibits dILP production under normal (non-dormancy) conditions^48,49^, and since dILPs can act as dormancy antagonists in flies^14,15^, an attractive hypothesis is that serotonin might promote dormancy by suppressing dILPs and thus by reducing IIS (Fig. 5).

To test this model we silenced the serotonin receptor (5-HT1A) under dormancy-inducing conditions specifically in the IPCs. In support of the idea that serotonin is required for dormancy by downregulating IIS, RNAi against *5-HT1A* in the IPCs substantially decreased dormancy and led to elevated expression of *dilp2* and *dilp5* but reduced expression of *4E-BP*, suggesting increased IIS activity. Abolishing serotonergic signaling in the IPCs is therefore sufficient to dramatically reduce dormancy levels, presumably due to the lack of inhibition of dILP production and release, thereby causing elevated IIS to prevent dormancy entry. Conversely, constitutive activation of serotonergic neurons markedly increased dormancy levels.

Our findings are consistent with the idea that serotonin promotes dormancy by decreasing dILP release, yet paradoxically, dormant wild type flies tend to exhibit increased *dilp* mRNA levels^14,15^. However, increased *dilp* levels might be due to compensatory upregulation of *dilp* expression in response to strongly reduced IIS as increased *dilp* expression levels often correlate with strongly decreased systemic IIS and with reduced release of dILPs from the IPCs^90,91^.

Our results thus provide causal evidence that serotonin signaling promotes adult reproductive dormancy in *Drosophila* (Fig. 5). Together with previous results from butterflies and moths, these findings suggest that serotonin might be a conserved dormancy/diapause agonist among insects.

In several insects, dopamine seems to play a key role in diapause entry and maintenance, with diapause-destined individuals exhibiting markedly elevated dopamine levels^33–36^. However, causal evidence for a direct role of dopamine in dormancy/diapause remains scarce, and the effects of dopamine on ovarian dormancy entry in *Drosophila* have not yet been investigated.

Our experiments provide five lines of evidence showing that dopamine acts as a major dormancy agonist in *Drosophila*. First, as in several diapausing insects, dormant fruit fly females have increased dopamine levels. Second, some *ple* and *Ddc* mutants exhibit reduced dopamine levels^74,92,93^; in line with this, we observed that a loss-of-function allele of *ple* and a *Ddc* hypomorph have strongly reduced levels of dormancy (*Ddc* is essential for both serotonin and dopamine synthesis; the failure of *Ddc*^hyp^ to undergo dormancy is thus consistent with a role of both neurotransmitters in dormancy) Third, *e*^1^ mutants with elevated dopamine^77^ show increased propensity to undergo dormancy. Fourth, constitutive activation of dopaminergic neurons strongly enhanced dormancy induction. Fifth, silencing of the dopamine receptor *DopR1* in the IPCs, the CA gland and in fat body, i.e. tissues critically important for dormancy physiology^1,17,83^, strongly decreased dormancy. Our results thus demonstrate that dopamine is a causally important promoter of dormancy.

In further support of a major role of dopamine signaling in dormancy, our assays showed that PKA signaling, downstream of DopR1 signaling, impacts dormancy entry: impairing the activity of PKA in the IPCs, CA and fat body substantially decreased the proportion of females able to enter dormancy. Impaired PKA activity specifically in the CA, the gland producing JH, led to decreased expression of *4E-BP* throughout the body, thus indicating increased IIS. Similarly, consistent with increased JH signaling, we observed that the levels of *Jon25Bii*, known to be induced by JH, are increased and that those of *obp99b*, typically downregulated by JH^25^, are decreased. Interestingly, our finding that reduced PKA activity in the CA decreases *4E-BP* in peripheral tissues might be consistent with observations suggesting that FOXO/IIS and JH are involved in a positive feedback loop, mutually regulating each other^89^. Our results thus lend support to a model whereby dopamine signaling promotes dormancy entry by modulating both IIS and JH signaling (Fig. 5). Since dopamine modulates diapause in animals as diverse as *C. elegans* and mammals^2,94^, dopamine signaling might represent an evolutionarily conserved (or co-opted) mechanism of dormancy/diapause regulation.

Since the expression and/or release of dILPs can be modulated by octopamine signaling (i.e., by OAMB^49,51^) we were also interested in determining whether octopaminergic signaling affects dormancy propensity. Contrary to expectation, however, RNAi directed against *OAMB* in the IPCs had no effect on dormancy, perhaps suggesting that octopamine does not regulate dormancy via modulating dILP levels. Yet, in marked contrast, constitutive activation of octopaminergic neurons decreased dormancy levels. Based on these results we conjecture that, unlike serotonin and dopamine, octopamine might represent a dormancy antagonist. However, an important caveat to this interpretation is that *dilp-2-3,5* mutants, which display elevated dormancy levels^15^, exhibit increased octopamine levels^95^. While the role of octopamine in dormancy physiology clearly requires further study, it is noteworthy that in *C. elegans* and flies, serotonin and dopamine often play opposing roles to octopamine in affecting behavior and neurophysiology, sometimes even mutually antagonizing each other^96–100^. We therefore hypothesize that such neurotransmitter antagonism might also be involved in the regulation of dormancy.

## Methods

### Fly stocks and maintenance

Prior to experiments fly stocks were maintained at 23 °C and a 12:12 hour light [L]: dark [D] cycle on a standard yeast-sucrose-cornmeal diet. We used the following fly stocks in our experiments (BDSC = Bloomington *Drosophila* Stock Center; VDRC = Vienna *Drosophila* RNAi center): (A) GAL4 driver lines: *Aug21-*GAL4 (BDSC #30137); *Cg-*GAL4 (=*collagen type IV* (*Col4a1*) = (*Cg*)*-*GAL4; BDSC #7011); *dilp2(p)-*GAL4 (courtesy of Eric J. Rulifson); *dilp2-*GAL4 (courtesy of Linda Partridge); *Gad1-*GAL4 (courtesy of Pierre Leopold); *hmgcr*^Di-11^*-*GAL4 (=*Di-11*-GAL4; courtesy of Jean-René Martin); *ppl-*GAL4 (courtesy of Michael J. Pankratz); *Tdc2-*GAL4 (BDSC #9313); *TH-*GAL4 (BDSC #8848); *Trh-*GAL4 (BDSC #38389); (B) UAS responder lines: UAS*-D2R-RNAi* (VDRC #11471); UAS*-DopR1-RNAi* (VDRC #107058); UAS*-GBR-RNAi* (VDRC #1784); UAS*-HA-Pka-R1.BDK33* (=*UAS-Pka-R33*; courtesy of Franḉois Rouyer); UAS*-5-HT1A-RNAi* (VDRC #106094); UAS*-NaChBac* (courtesy of Michael O’Connor); UAS*-OAMB-RNAi* (VDRC #2861); UAS-*Pka-R1.BDK35* (=*UAS-Pka-R35*, BDSC #35550); UAS*-Upd2* (courtesy of Norbert Perrimon); (C) Mutant lines: *Ddc*^DE1^ (=*Ddc*^hyp^, BDSC #3168); *dumb*^3^ (=*DopR1* hypomorph = *DopR1*^hyp^, BDSC #19491); *e*^1^ (BDSC #1658); *ple*^4^ (BDSC #3279); *w*^1118^(*ls-tim*); and *w*^1118^*(s-tim)* (courtesy of Charlotte Helfrich-Föster).

### Genetic controls and genetic background

Since SNPs at the *timeless (tim)* and *couch potato* (*cpo)* loci can affect dormancy^13,101,102^, we sought to control for potentially confounding effects of these SNPs in our experiments by determining their allelic states in all the stocks used. To genotype the *tim* locus we extracted genomic DNA from 5–10 females from each stock. Homogenates were incubated at 37 °C for 45 min in 50 μL of solution A (Tris HCl pH 8.2 10 mM, EDTA 2 mM, NaCl 25 mM), adding 1 μL proteinase K (10 mg/mL), followed by 3 min at 100 °C. We used amplification refractory mutation system (ARMS) PCR^13^, with the following primers: *ls-tim* forward: 5′-TGGAATAATCAGAACTTTGA-3′; *s-tim* forward: 5′-TGGAATAATCAGAACTTTAT-3′; *s-tim* reverse: 5′-AGATTCCACAAGATCGTGTT-3′ (common). To determine the *cpo* genotype of experimental strains, we collected 5 individuals from each strain employed for dormancy assays and extracted genomic DNA. The region encompassing the *cpo*^A347V^ and *cpo*^48034(A/T)^ SNPs^101,102^ was amplified via PCR. Templates were sequenced after purification in minicolumns (Wizard® SV Gel and PCR Clean-Up System). We used the following primers: forward 5′-AACATCCGTTGCTGCTGTC-3′; reverse 5′-CCCCAAGCTGTCACTTTTGT-3′.

Although the “high-dormancy” allele *cpo*^347V^^102,103^ was found to be present in homozygous state in the *ple*^4^/+ strain, this mutant exhibits a very low level of dormancy, so that the *cpo* SNP is unlikely to have a confounding effect on our results for *ple*^4^ (see Supplementary Table S1). All other experimental genotypes (e.g. F1 of GAL4 × UAS crosses), made from crosses with a heterozygous *cpo*^347A/V^ (UAS or GAL4) parent, had segregating *cpo*^347V^ alleles, but there is no evidence that the heterozygous combination of these *cpo* alleles affects dormancy (see Supplementary Table S1). In contrast, the potentially confounding *ls-tim/s-tim* polymorphism was highly prevalent in our stocks; we thus always compared experimental and control genotypes with matching *tim* alleles; thus, our experimental comparisons are not confounded by the *tim* polymorphism (see Supplementary Table S1).

For GAL4 > UAS experiments we used the corresponding GAL4 and UAS lines crossed to *w*^1118^ as controls. For experiments using mutants, we generally used *w*^1118^ as a generic “wild type” control since the majority of the mutant strains used has been made in that background; depending on the *tim* genotype of the experimental F1 genotypes, we either used *w*^1118^ (*s-tim*) or *w*^1118^(*ls-tim*) for control crosses (see above). Note that mutant phenotypes also differed significantly in their dormancy levels from other “control” genotypes, e.g. controls used in GAL4 > UAS assays and carried in parallel to the mutant assays (data not shown).

Many of our experiments were carried out in parallel and thus used the same internal genetic controls; for the sake of clarity we often display distinct results that involve the same controls (i.e., identical values for the controls) in separate figures. The following list states which identical controls are shown (replotted) in different contexts: *dilp2*-GAL4/+ (ls) (Figs 1A,G,I and 3A); *dilp2(p)*-GAL4/+ (ls) (Supplementary Figure S1A-C and Supplementary Figure S2A); UAS-*5HT1A*-RNAi/+ (Fig. 1A and Supplementary Figure S1A); UAS-*NaChBac*/+ (Fig. 1B,H,J); UAS-*OAMB*-RNAi/+ (Fig. 1G and Supplementary Figure S1B); UAS-*GBR*-RNAi/+ (Fig. 1I and Supplementary Figure S1C); UAS-*Upd2*/+ (Fig. 1K and Supplementary Figure S1D); UAS-*DopR1*-RNAi/+ (set 1: Fig. 3A and Supplementary Figure S2A); UAS-*DopR1*-RNAi/+ (set 2: Fig. 3B,C and Supplementary Figure S2B,C); UAS-*D2R*-RNAi/+ (Fig. 3B,C) and Supplementary Figure S2B,C); UAS-*PKA-R33*/+ (set 1: Fig. 3D and Supplementary Figure S2D); UAS-*PKA-R33*/+ (set 2: Fig. 3E-F) and Supplementary Figure S2E-F); UAS-*PKA-R35*/+ (Fig. 3E-F) and Supplementary Figure S2E-F).

We note that most of the experimental controls, independent of their precise identity, showed qualitatively very similar values of dormancy incidence.

### Ovarian dormancy assays

To identify positive regulators of ovarian dormancy, we used RNAi constructs (or mutants) under dormancy-inducing conditions (low temperature, short photoperiod), seeking cases of significantly decreased dormancy silencing positive regulators is expected to reduce or block dormancy under dormancy-permissive conditions. In a few cases, i.e. for manipulations expected to increase dormancy, we performed assays under long-day conditions (16 h:8 h L:D): long days can inhibit dormancy (cf.^104^; but also see^105,106^; reference^106^ in particular shows that dormancy in *D. melanogaster* is predominantly elicited by temperature), thus providing a conservative estimate of dormancy propensity. This was done for all assays involving the activation of aminergic neurons with UAS-*NaChBac* and the *e*^1^ mutant (and corresponding controls).

Prior to dormancy assays, larvae were reared under standard conditions at 23 °C and 12 h:12 h L:D until eclosion. Newly eclosed virgin flies were collected (~60 females and 60 males per replicate) within 5 hours of eclosion and subsequently exposed to low temperature (12 °C) and short photoperiod (8 h:16 h L:D) for 11 days. Female ovarian dormancy was scored as the complete absence of vitellogenic oocytes (i.e., all oocytes at stages ≤ 7) by examining all ovarioles in both ovaries of each fly^11,13,101^. Between 4–7 replicates (~60 females each, ~300 flies in total) were assayed per genotype. In the figures, dormancy levels are shown as the percentage of dormant females ± binomial standard error (SE, in %). Percentage dormancy data were arcsine square-root transformed and analyzed with ANOVA (followed by Tukey post-hoc tests) in R (v.2.15.1); figures in the Results section show untransformed data.

### qRT-PCR assays

mRNA was isolated from whole bodies (for *4E-BP* we used thoraces and abdomens), generating 4 replicates per genotype, each consisting of 10–14 females; for *dilps* we extracted mRNA from 25 heads per replicate, with 3 replicates per genotype. mRNA was reverse-transcribed with SuperScript II First-Strand Synthesis SuperMix (Invitrogen). PCRs were performed on a 7500 Real Time PCR System (Applied Biosystems), with GoTaq qPCR Master Mix (Promega) and the following primers: *dilp2* F: 5′-ATCCCGTGATTCCACACAAG-3′, R: 5′-GCGGTTCCGATATCGAGTTA-3′; *dilp3* F: 5′-CCGAAACTCTCTCCAAGCTC-3′, R: 5′-GCCATCGATCTGATTGAAGTT-3′; *dilp5* F: 5′-GCCTTGATGGACATGCTGA-3′, R: 5′-CATAATCGAATAGGCCCAAGG-3′; *4E-BP* F: 5′-TACACGTCCAGCGGAAAGTT-3′, *R*: 5′-CCTCCAGGAGTGGTGGAGTA-3′; *obp99b* F: 5′-AGCACGGATTCGATGTCCACAAGA-3′, R: 5′-TTGGAGTTCATGAAGCACATGCCG-3′; *Jon25Bii* F: 5′-CAGGCTCAGTACACCCACAC-3′, R: 5′-TGGTGTTGTAGTCCGAGTGC-3′; *rp49* F: 5′- ATCGGTTACGGATCGAACAA-3′, R: 5′- GACAATCTCCTTGCGCTTCT-3′. mRNA levels were normalized to the levels of the control gene *rp49* by using the 2^−ΔΔCT^ method. Data were analyzed with ANOVA (followed by Tukey post-hoc tests) in R (v.2.15.1).

### Dopamine quantification

To quantify dopamine levels we used *dilp2*-GAL4/+ as a “wildtype”, with flies being exposed for 11 days to either dormancy (either 12 °C and 8 h:16 h L:D or 12 °C and 12 h:12 h L:D) or control (non-dormancy) conditions (23 °C and 12 h:12 h L:D). Three replicates of 500 females each were homogenized in ice-cold 0.1 M HClO_4_, samples centrifuged at 13.000 rcf for 10 min supernatants filtered with Minisart® 0,45 µm filters. For dopamine quantification we used a Chromosystems catecholamine determination kit. Prior to chromatographic analysis samples were prepared as follows: 100 µL of internal standard and 6 mL of neutralization buffer were added to 3 mL of sample to adjust pH. Next, samples were purified with sample clean up columns, washed first with water and then with elution buffer. Subsequently, dopamine levels were measured with HPLC-ECD. 20 µL of eluted sample were injected in a C18 reverse-phase column (Chromsystems). Data were acquired with Geminyx software (Chromsystems) and analyzed with *t*-tests.

## Electronic supplementary material

Supplementary Information
