# Supplementary material for: Aminergic Signaling Controls Ovarian Dormancy in Drosophila
Source: Sci Rep. 2018 Feb 1;8:2030. doi: 10.1038/s41598-018-20407-z (PMC5794970; doi:10.1038/s41598-018-20407-z)
Supplement: Supplementary file 1 — Supplementary Information [file 41598_2018_20407_MOESM1_ESM.pdf]

Supplementary Information

Aminergic Signaling Controls Ovarian Dormancy in *Drosophila*

Gabriele Andreatta[1], Charalambos P. Kyriacou[2], Thomas Flatt[3,4]*, and Rodolfo Costa[1]*

[1]Department of Biology, University of Padova, Padova, Italy

[2]Department of Genetics, University of Leicester, Leicester, United Kingdom

[3]Department of Ecology and Evolution, University of Lausanne, Lausanne, Switzerland

[4]Department of Biology, University of Fribourg, Fribourg, Switzerland

*Corresponding authors: rodolfo.costa@unipd.it, thomas.flatt@unifr.ch

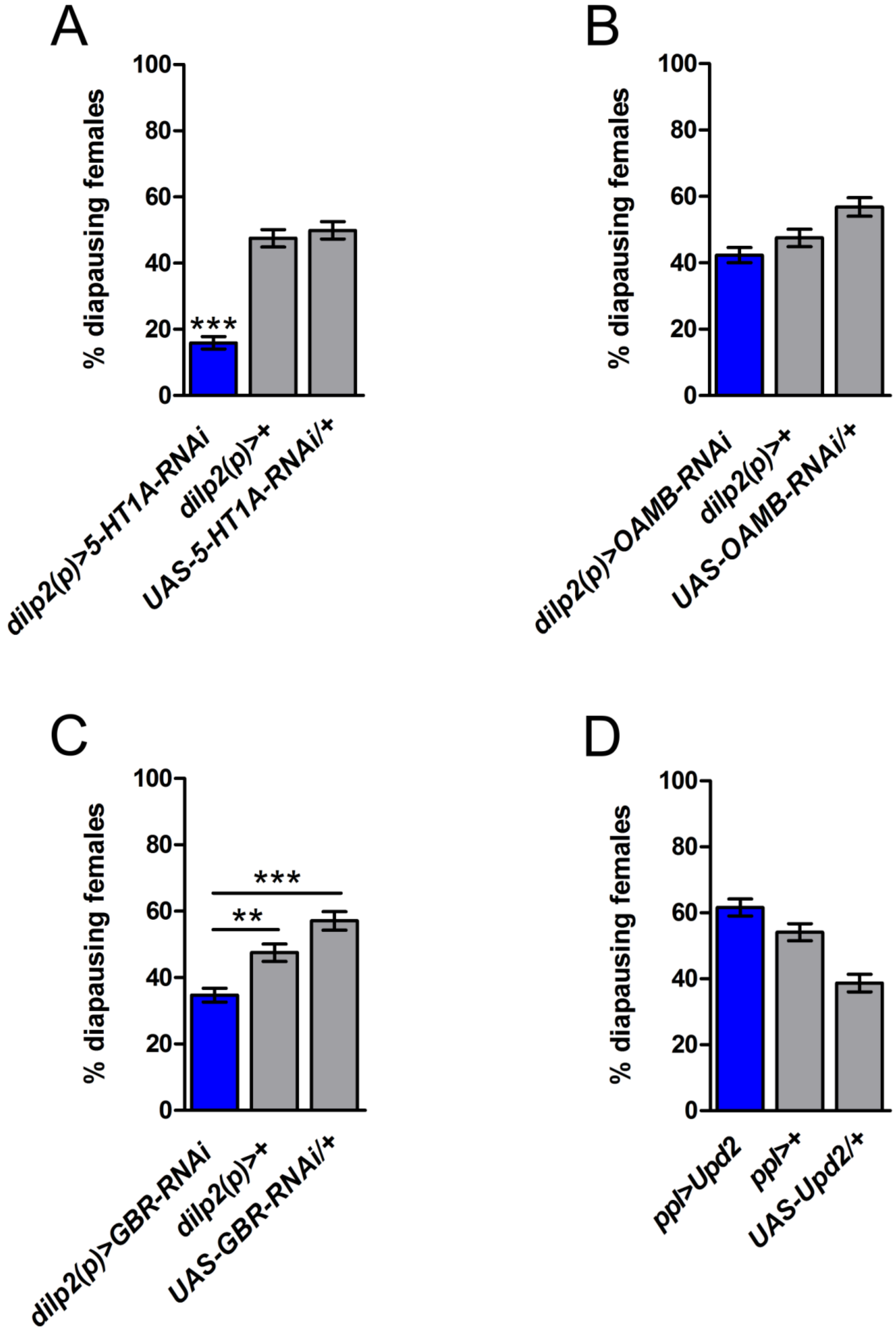

Fig. S1. Downregulation of serotonin receptor in the IPCs markedly impairs dormancy, but silencing of *OAMB* and *GBR* has little or no effect. (A) Knockdown of the serotonin receptor (*5-HT1A*) in the IPCs using *dilp2(p)*-GAL4

markedly reduces dormancy diapause (also see Fig. 1A and B). (B) In contrast, silencing octopaminergic signaling via downregulation of *OAMB* in the IPCs with *dilp2(p)*-GAL4 does not affect dormancy (also see Fig. 1G and H). (C) RNAi knockdown of *GBR* in the IPCs with *dilp2(p)*-GAL4 causes a moderate reduction of ovarian dormancy; however, the results in Figure 1J suggest that this effect may not be consistent and general. (E) Overexpression of *Upd2* in fat body with *ppl*-GAL4, a manipulation that blocks GABA-mediated inhibition of dILP release from the IPCs, does not affect dormancy (also see Fig. 1K). Figures show dormancy levels as the percentage of females in dormancy (mean ± binomial SE); each assay was performed with 5-7 replicates per genotype, each replicate consisting of ~60 females. $^{**}p<0.01$; $^{***}p<0.001$.

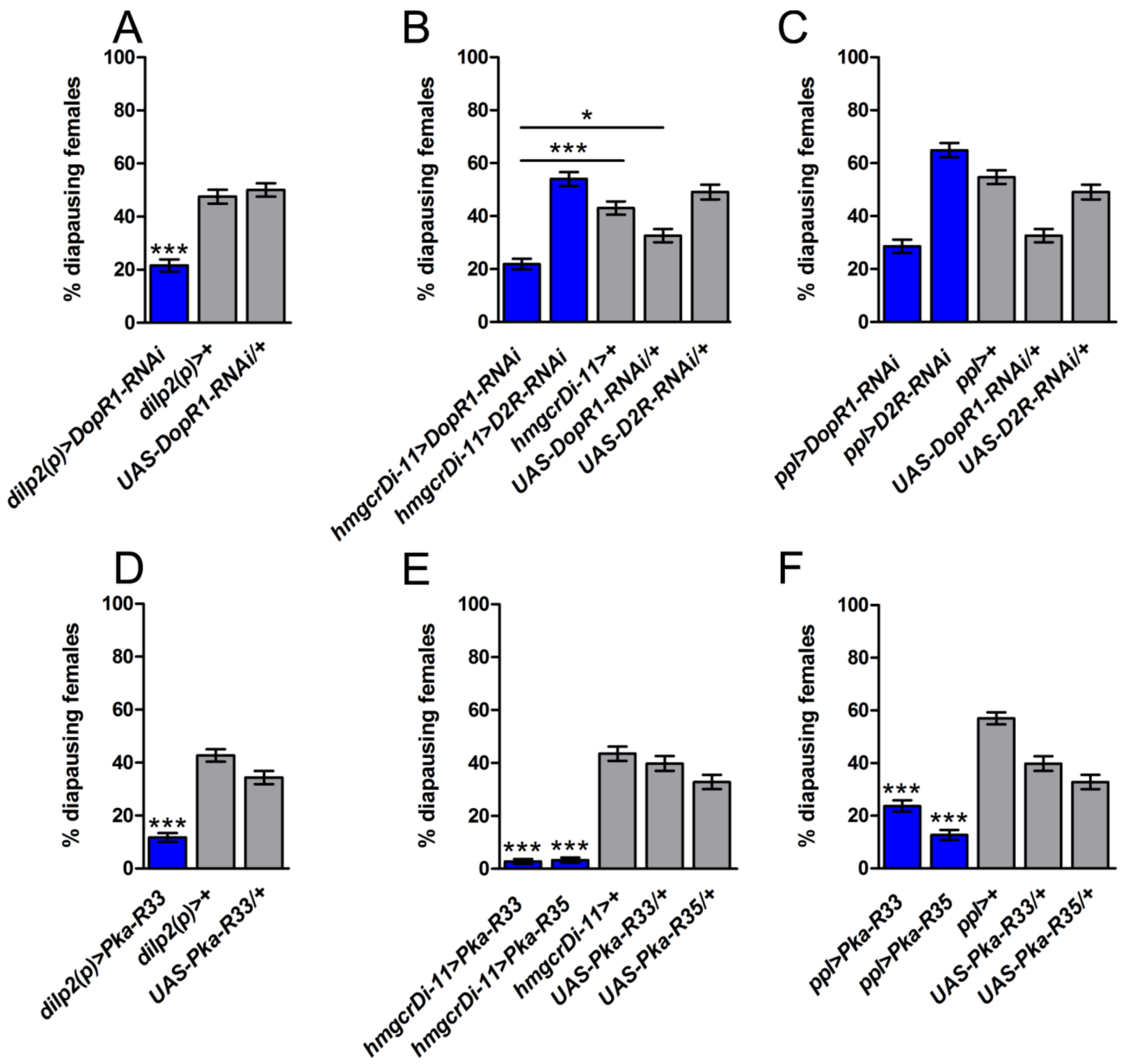

Fig. S2. Downregulation of DopR1 and PKA signaling in IPCs, CA and fat body reduces dormancy response. (A) Knockdown of *DopR1* in the IPCs with *dilp2(p)*-GAL4 reduces dormancy levels (cf. Figure 3A). (B-C) In CA (B) and fat body (C), downregulation of *DopR1* but not of *D2R* decreases dormancy (also see Figure 3B and C). (D-F) Impairing PKA signaling in IPCs, CA and fat body dramatically reduces dormancy (also cf. Figure 3D-F). Shown are percentages of dormant females (mean ± binomial SE); each assay was performed with 5-7 replicates per genotype, each replicate consisting of ~60 females. *p<0.05; ***p<0.001.

Genotype	Figure	L:D	% dormancy ± SE	*cpo*^{A347V}	*cpo*^{48034} (A/T)	***Tim*** **(s/ls)**
*dilp2>5-HT1A-*RNAi	1A	8:16	11.7 ± 1.3	*V/A*	A/A	s/ls
dilp2>+	1A, 1G, 1I, 3A	8:16	37.5 ± 2.6	V/A	A/A	s/ls
UAS-*5-HT1A-*RNAi/+	1A, S1A	8:16	49.9 ± 2.6	*A/A*	A/A	ls/s
Trh>NaChBac	1B	16:8	87.5 ± 1.7	*A-V/A*	A/A	s/s
Trh>+	1B	16:8	61.5 ± 2.7	A-V/A	A/A	s/s
UAS-*NaChBac/+*	1B, 1J	16:8	50.2 ± 2.7	*A/A*	A/A	s/s
*dilp2>OAMB-*RNAi	1G	8:16	31.3 ± 2.6	V/A	A/A	s/ls
UAS-*OAMB-*RNAi/+	1G, S1B	8:16	56.8 ± 2.8	A/A	A/A	ls/s
Tdc2>NaChBac	1H	16:8	26.0 ± 1.9	A/A	A/A	ls/s
Tdc2>+	1H	16:8	44.7 ± 2.3	A/A	A/A	ls/s
UAS-*NaChBac/+*	1H	16:8	59.5 ± 2.8	*A/A*	A/A	s/ls
dilp2>GBR-RNAi	1I	8:16	29.9 ± 1.8	*V/A*	A/A	s/ls
UAS-*GBR-*RNAi/+	1I, S1C	8:16	57.1 ± 2.8	*A/A*	A/A	ls/s
Gad1>NaChBac	1J	16:8	46.0 ± 2.8	V/A	A/A	s/s
Gad1>+	1J	16:8	61.9 ± 2.8	V/A	A/A	s/s
cg>Upd2	1K	8:16	41.0 ± 2.7	A/A	A/A	s/s
cg>+	1K	8:16	52.4 ± 2.6	A/A	A/A	s/s
UAS-*Upd2/+*	1K, S1D	8:16	38.7 ± 2.7	A/A	A/A	s/s
ple^{4}*/+*	2B	8:16	12.0 ± 1.5	V/V	A/A	s/s
Ddc^{hyp}	2B	8:16	6.4 ± 1.5	A/A	A/A	ls/ls
DopR1^{hyp}	2B	8:16	21.8 ± 1.7	A/A	T/T	s/s
e^{1}	2B	16:8	88.1 ± 1.4	A/A	A/A	ls/ls
w^{1118} *(s-tim)*	2B	8:16	39.2 ± 2.4	A/A	A/A	s/s
w^{1118} *(ls-tim)*	2B	8:16	57.6 ± 1.8	A/A	A/A	ls/ls

w[1118] *(ls-tim)*	2B	16:8	51.6 ± 2.0	A/A	A/A	ls/ls
TH>NaChBac	2D	16:8	87.8 ± 1.4	A/A	A/A	s/s
TH>+	2D	16:8	70.1 ± 2.0	A/A	A/A	s/s
UAS-*NaChBac/+*	2D	16:8	42.4 ± 2.5	A/A	A/A	s/s
*dilp2>DopR1-*RNAi	3A	8:16	9.3 ± 1.6	V/A	A/A	s/ls
UAS-*DopR1-*RNAi/+	3A, S2A	8:16	50.0 ± 2.5	A/A	A/A	ls/s
*Aug21>DopR1-*RNAi	3B	8:16	13.2 ± 1.9	A/A	A/A	s/ls
*Aug21>D2R-*RNAi	3B	8:16	62.7 ± 2.6	A/A	A/A	s/ls
Aug21>+	3B	8:16	61.7 ± 2.8	A/A	A/A	s/ls
UAS-*DopR1-*RNAi/+	3B, 3C, S2B, S2C	8:16	32.6 ± 2.5	A/A	A/A	ls/s
UAS-*D2R-*RNAi/+	3B, 3C, S2B, S2C	8:16	49.1 ± 2.8	A/A	A/A	ls/s
cg>DopR1-RNAi	3C	8:16	3.5 ± 1.0	A/A	A/A	s/ls
cg>D2R-RNAi	3C	8:16	32.6 ± 2.7	A/A	A/A	s/ls
cg>+	3C	8:16	38.8 ± 2.8	A/A	A/A	s/ls
dilp2>Pka-R33	3D	8:16	5.8 ± 1.3	V/A	A/T	s/s
dilp2>+	3D	8:16	34.6 ± 2.4	V/A	A/A	s/s
UAS-*Pka-R33/+*	3D, S2D	8:16	34.4 ± 2.5	A/A	A/A	s/s
Aug21>Pka-R33	3E	8:16	0.3 ± 0.3	A/A	A/A	s/s
Aug21>Pka-R35	3E	8:16	0.3 ± 0.3	A/A	A/T	s/s
Aug21>+	3E	8:16	69.5 ± 2.4	A/A	A/A	s/s
UAS-*Pka-R33/+*	3E, 3F, S2E, S2F	8:16	39.8 ± 2.8	A/A	A/A	s/s
UAS-*Pka-R35/+*	3E, 3F, S2E, S2F	8:16	32.8 ± 2.7	A/A	T/A	s/s
cg>Pka-R33	3F	8:16	10.1 ± 1.5	A/A	A/A	s/s
cg>Pka-R35	3F	8:16	5.4 ± 1.2	A/A	A/T	s/s
cg>+	3F	8:16	46.4 ± 2.8	A/A	A/A	s/s

dilp2(p)>5-HT1A-RNAi	S1A	8:16	15.9 ± 1.9	*A/A*	A/A	s/ls
dilp2(p)>+	S1A, S1B, S1C, S2A	8:16	47.5 ± 2.6	A/A	A/A	s/ls
dilp2(p)>OAMB-RNAi	S1B	8:16	42.3 ± 2.3	A/A	A/A	s/ls
dilp2(p)>GBR-RNAi	S1C	8:16	34.7 ± 2.1	*A/A*	A/A	s/ls
ppl>Upd2	S1D	8:16	61.6 ± 2.6	A/A	T/A	s/s
ppl>+	S1D	8:16	54.1 ± 2.6	A/A	T/A	s/s
dilp2(p)>DopR1-RNAi	S2A	8:16	21.6 ± 2.3	A/A	A/A	s/ls
$hmgcr^{Di-11}$*>DopR1*-RNAi	S2B	8:16	21.9 ± 2.0	V/A	A/A	s/ls
$hmgcr^{Di-11}$*>D2R*-RNAi	S2B	8:16	54.0 ± 2.6	V/A	A/A	s/ls
$hmgcr^{Di-11}$*>+*	S2B	8:16	43.0 ± 2.5	V/A	A/A	s/ls
ppl>DopR1-RNAi	S2C	8:16	28.6 ± 2.5	A/A	T/A	s/ls
ppl>D2R-RNAi	S2C	8:16	64.9 ± 2.7	A/A	T/A	s/ls
ppl>+	S2C	8:16	54.7 ± 2.6	A/A	T/A	s/ls
dilp2(p)>Pka-R33	S2D	8:16	11.7 ± 1.7	A/A	A/A	s/s
dilp2(p)>+	S2D	8:16	42.7 ± 2.3	A/A	A/A	s/s
$hmgcr^{Di-11}$*>Pka-R33*	S2E	8:16	2.8 ± 0.9	V/A	A/A	s/s
$hmgcr^{Di-11}$*>Pka-R35*	S2E	8:16	3.3 ± 1.0	V/A	A/T	s/s
$hmgcr^{Di-11}$*>+*	S2E	8:16	43.5 ± 2.7	V/A	A/A	s/s
ppl>Pka-R33	S2F	8:16	23.7 ± 2.2	A/A	T/A	s/s
ppl>Pka-R35	S2F	8:16	12.7 ± 1.9	A/A	T/T	s/s
ppl>+	S2F	8:16	57.0 ± 2.3	A/A	T/A	s/s

Table S1. Summary of experimental data. The first four columns indicate the name of the experimental line / genotype used, the figures where the dormancy results are shown, the photoperiod used for the assay, and the average dormancy percentage ±

binomial standard error (SE, in %). The last three columns give information on the genetic background at the *cpo* and *tim* loci for the lines used. For the cpo^{A347V} SNP, the two different alleles/codons encode Alanine (A) or Valine (V); for the $cpo^{48034(A/T)}$ SNP, we indicate the presence of Adenine (A) or Thymine (T); for the *tim* locus, the alleles are designated as s = short or ls = long-short. For further details see Results.
